# Feasibility of smartphone-supported, combined physical and cognitive activities in the Neighbourhood for stimulating social participation of the elderly

**DOI:** 10.1186/s12877-022-03303-0

**Published:** 2022-07-30

**Authors:** Christian Thiel, Liane Günther, Anke Osterhoff, Sascha Sommer, Christian Grüneberg

**Affiliations:** 1 Hochschule für Gesundheit (University of Applied Sciences), Department of Applied Health Sciences, Division of Physiotherapy, Gesundheitscampus 6-8, 44801 Bochum, Germany; 2 Faculty of Sports Science, Training and Exercise Science, Ruhr-University, Bochum, Germany; 3 Institute of Medical Sociology, Centre for Health and Society, Medical Faculty, University of Düsseldorf, Moorenstraße 5, 40225 Düsseldorf, Germany; 4 Hochschule für Gesundheit (University of Applied Sciences), Department of Applied Health Sciences, Speech and Language Therapy Program, Gesundheitscampus 6-8, 44801 Bochum, Germany

**Keywords:** Combined cognitive and physical activity, Smartphone support, Neighbourhood, Social participation, Community-dwelling adults

## Abstract

**Background:**

Combining smartphone-assisted group activities in the neighbourhood and training in physical and cognitive skills may offer the potential to promote social participation and connectedness of older adults. This non-controlled proof-of-concept, retrospectively registered study aimed to determine the feasibility of such an intervention approach, including its evaluation.

**Methods:**

In two consecutive six-month intervention cycles, 39 community-dwelling adults were provided with weekly smartphone, physical and cognitive training by two tutors. Using a specifically designed app, the participants were also encouraged to join and later self-organise physically and cognitively stimulating activities related to hot spots in their Bochum neighbourhood. Indicators of feasibility were documented.

**Results:**

The recruitment and assessments took 3 hours per participant. Excluding smartphone support, the preparation and the implementation of the intervention amounted to nine person-hours per week.

Six participants dropped out, and 13 did not complete one or more assessments. The participants attended 76 ± 15% of the weekly training sessions. The instructors deemed the programme feasible, but familiarisation with the smartphone and the app was very time-consuming.

Twenty-seven of 29 participants reported high overall satisfaction, and 22 agreed that the programme helped them to establish social contacts. The smartphones attracted substantial interest and were used frequently, despite mixed satisfaction with the project-specific app. From baseline to follow-up, the six-minute walking distance, lower extremity strength and moderate to vigorous physical activity, as well as quality of life, were preserved at a high level, while balance performance was significantly improved. Of the 11 tests related to cognitive functioning, 4 tests (a memory test, the Stroop test and 2 tests of verbal fluency) indicated significant improvement. No moderate or serious adverse events occurred in relation to the assessments or the intervention.

**Conclusions:**

The multimodal approach seems safe and feasible and offers the potential to promote social connectedness, bonds in the residential neighbourhood and smartphone competency, as well as to preserve or improve physical and cognitive functions. Adaptations of the intervention and of the outcome assessments may contribute to better assessment and exploitation of the potential of this approach in a future study involving socially, physically and cognitively less active elderly persons.

## Background

### Social participation and the role of the neighbourhood in the ageing population

Most older adults like to engage in social activities [1]. Pursuing meaningful activities in a community and cultivating relationships may indeed constitute an important component of successful ageing [2]. However, later life often comes with decreased physical and cognitive performance and deteriorating health [3], which might reduce social engagement [4]. The relations between social participation, physical and cognitive functions, and health in the ageing population are likely complex and bidirectional and may operate through multiple pathways [5, 6].

While social participation is of high relevance to older adults, the proportion of older people living alone has increased worldwide [7]. The family structure and living arrangements have fundamentally changed, providing less informal opportunities for social interaction and increasing the risk of disability in basic activities of daily living [8], as well as of deteriorating physical and mental health [9]. In this context, the neighbourhood has become more important as a framework for social relationships and participation in society [10]. At the same time, older adults identify with the neighbourhood as their residential environment and wish to remain in their own homes as long as possible [11].

The constituents of this neighbourhood may vary, and definitions can operate on various dimensions (e.g., geographical, physical, administrative, perceptual and activity-related). According to Rüßler and Stiel [12], a neighbourhood can be regarded as a social environment that emerges from social interaction, is socially formed, has manifold social functions, is easily graspable and primarily relates to daily life, has an impact on its inhabitants’ perceptions and actions, and offers the potential for identification and empathy.

Interventions that directly or indirectly contribute to ageing individuals’ ability to maintain or expand their social participation and social relations in their neighbourhood will likely improve the conditions and the quality of their lives [10]. Physically and cognitively stimulating activities that involve group interaction and are related to daily life in the neighbourhood may offer opportunities for social interaction, as well as promote its physical and cognitive prerequisites. When designing programmes that provide such interventions, the needs and preferences of the individuals involved, as well as their view on participation issues, should be considered [13, 14]. To increase their appeal and accessibility, such group-based programmes should be complemented with individual support and training elements, reflecting the older age group’s enormous heterogeneity in their constitutions, needs and habits [8].

### Physical and cognitive activities and training for social participation

Physical activity may be a meaningful leisure pursuit and group activity that can be done in the neighbourhood. Equally important, it also helps reduce age- and health-related impairments in everyday life and slows down or even reverses the decrease in strength, endurance and flexibility. Regular activities, such as walking, dancing or light gardening work [15, 16], may improve physical performance, function and mobility. Specific exercise training may trigger greater benefits, as long as its form, intensity, duration and frequency are individually adapted to the participants’ physical functions and needs [17–19].

Exercise is also effective for older people with mobility limitations [20]. Several training programmes, such as the Otago Exercise Program, Weight-Bearing Exercise for Better Balance (WEBB), Functional Task Exercise (FUNTEX), High Intensity Functional Exercise (HIFE) and Lifestyle Functional Exercise Program (LIFE), continue to spread internationally because of their good scientific evidence [21–23], including economic benefits related to fall prevention in some cases (Otago, WEBB and LIFE) [24–26]. Nonetheless, transfer effects on everyday mobility (in the sense of distance covered and radius of action) and even more importantly, on social participation, are still unclear. Physical activities are generally regarded as more enjoyable if done in a group [27], which may result in new social connections. However, a review and meta-analysis of 16 randomized trials with 2315 participants presenting with a wide range of chronic diseases and mobility limitations shows that physical activity per se does not automatically improve older people’s participation in social life (SMD = 0.03; 95% CI = − 0.10 to 0.16) [28]. Only exercise interventions lasting 12 months or longer were slightly beneficial (SMD = 0.15; 95% CI = 0.02 to 0.28). In their conclusion, the authors argue for the development of more complex interventions which go beyond physical activity, and which address both participation and its various determinants [28].

Cognitive training is another intervention that can be organised as a meaningful group activity and can be related to the neighbourhood. There is sufficient evidence of the effects of cognitive training on outcomes such as neuroplastic changes in the brain assessed by neuroimaging [29], patient self-esteem [30] or executive function [31]. The effects are larger for task-specific skills, that is, if the exercise closely matches the target real-life task [32, 33]. Cognitive training can enhance the management of the tasks that are relevant for participation in everyday life. For instance, the training in cognitive processing speed can improve the management of instrumental activities of daily life, such as shopping, using public transportation, managing finances and taking responsibility for one’s own medication [34], as well as gait performance [35].

When interlaced in multimodal programmes, physical and cognitive activities or training promise better effects on the functions with high relevance for social participation than solitary interventions [36]. Programmes such as ‘Maintaining and supporting Independent Living in Old Age’ (SimA) [37] or ‘NeuroVitalis’ [38] provide evidence-based approaches to cognitive training for older people. SimA is a multimodal programme including competence training (general strategies of coping with age-related changes and everyday problems of the elderly), and memory training. SimA has shown long-lasting effects in domains that are important for independent living, as well as actual (self- and externally assessed) independence [37]. NeuroVitalis, another cognitive training programme aimed at older people, has shown small but promising effects [39–41]. It includes exercises to improve focus and attention, memory, and executive function. Both SimA and NeuroVitalis involve individual as well as dialogue-based and group exercises at various levels of difficulty, and can be complemented by physical training. Among stroke patients, cultural and social activities that comprise physical and cognitive stimulation, supplemented by specific individual support for physical and cognitive fitness, have been shown to contribute to the preservation and promotion of autonomy and participation [42].

Exercise training and a physically, intellectually and socially active lifestyle also contribute to the prevention or the delayed onset of a range of cardiovascular, metabolic, neoplastic and mental diseases [43, 44].

### Digital support for participation

Digital information and communication systems may facilitate social participation related to physical and cognitive activities in the neighbourhood [45, 46]. Smartphones or tablets offer community functions, as well as access to social media and to information on events in the neighbourhood. Such devices may also provide general information on health-enhancing behaviour and physical or cognitive exercises, as well as individual activity tracking [47]. According to a survey among the German-speaking population released in November 2020, 82% of Germans aged 60-69 years, and 52% of Germans 70 years and older have used a smartphone at least from time to time [48].

However, choosing the appropriate device, application and setup may pose a significant obstacle for technically inexperienced people [49]. Discovering the functions and learning how to handle a tablet or a smartphone are cognitively stimulating, but the familiarisation process may require considerable time and motivation [50, 51]. Among other factors, complex menu navigation, small font sizes and the requirement for fine-tuned movements can present further barriers to tablet or smartphone use by older people [49–51] 

Taken together as a simplified theory of change, combining technology-assisted group activities and training in physical and cognitive functions in the neighbourhood may offer various pathways to promote social participation and connectedness of older adults. Such a multimodal programme might include various components [52] and adhere to certain principles, as follows:

1. *Provide and enable social interaction.* The intervention should be aimed at stimulating social interaction, as well as enhancing cognitive and physical resources that are relevant for everyday life and social participation [53, 54].
2. *Take place in the neighbourhood.* Activities should be related to the neighbourhood, based on what the participants perceive as their hot spots, that is, nearby places to which they can relate and that are meaningful to them [55, 56].
3. *Involve participants.* While supported by instructors throughout the programme, the participants should be invited to contribute to and act as co-producers of the interventions [57].
4. *Use digital resources.* The participants should carefully be introduced and have easy access to new digital devices and technologies (e.g., smartphones and specific apps) [52].

Such multimodal approaches are rare and have scarcely been tested scientifically.

## Objectives

This proof-of-concept study focuses on the promotion of social participation of older people without or with only slight physical and cognitive impairments. It aims to test whether the components and the principles listed in the preceding section could be included and adhered to in a comprehensive programme called *Quartier Agil* (literally, Agile Quarter), as well as to determine the magnitude of potential effects in two exploratory intervention cycles. While analysing the programme’s feasibility [58], the following aspects should be covered:

1. Report the resources necessary for the recruitment and assessments, as well as for the implementation of the intervention’s various components.
2. Report the arrangement of the intervention, and analyse participant and instructor satisfaction, as well as retention rate. Report important lessons learned during the process of setting up *Quartier Agil* (problems that occurred, adaptations made, solutions found).
3. Reflect on the adequacy of the outcome parameters.
4. Estimate the possible magnitude of the effects on social participation, physical function, physical activity and cognitive-linguistic performance and functionality, as well as the safety of the intervention (adverse events).
5. Decide whether a larger study, which can more comprehensively investigate the *Quartier Agil* approach, seems warranted, as well as whether there should be changes to the study setup, intervention or assessments.

## Methods

This non-controlled proof-of-concept study comprised a preparation period and two six-month intervention cycles (January to June 2017, and August 2017 to January 2018) in a selected neighbourhood. For each intervention cycle, the plan was to recruit 20 participants by placing advertisements in local newspapers, distributing flyers and putting up posters on relevant sites of the neighbourhood (e.g., churches, cafés frequented by seniors), as well as to solicit the support of community stakeholders in social services and charitable institutions.

No sample size calculation was performed. No interim analyses were planned, and no stopping guidelines were in place. After study commencement, apart from a pre-planned refinement of the intervention after the first cycle, no further changes were made to the study design, methods or outcomes.

The inclusion criteria were as follows:

- community-dwelling in the selected neighbourhood,
- minimum age of 63 years and
- written declaration of consent (> 24 hours after verbal and written information).

The exclusion criteria were as follows:

- receiving home care or residential care,
- absolute contraindications regarding physical activity or exercise, according to the American College of Sports Medicine [59] or the German Society of Sports Medicine and Prevention [60], including, but not limited to, significant cardiac diseases such as decompensated heart failure, unstable angina, critical aortic stenosis, resting systolic BP > 200 mmHg, or resting diastolic BP > 110 mmHg; cancer with extreme nausea, fatigue or severe hematologic condition (such as thrombocytes < 20.000 μl); severe neurologic conditions such as ataxia; acute systemic illness or fever; uncontrolled type 2 diabetes or type 2 diabetes with severe foot ulcers; end-stage pulmonary disease such as COPD GOLD 4; musculoskeletal conditions that would prohibit exercise such as a severe rheumatoid arthritis flare; and
- cognitive deficits exceeding a mild cognitive impairment (ICD-10-Code: F06.7) as assessed by the Montreal Cognitive Assessment (MoCA) [61, 62]

No exclusion criteria relating to social interaction and cognitive or physical activity were defined. For this exploratory study, it was deliberately accepted that the participants were volunteers who might already show a high level of activity in any of these domains, thereby possibly diminishing potential intervention effects. The research team hoped that these volunteers would become better co-producers of the multimodal intervention and might also be able to better assist other (less active and experienced) group members. Further, participants with high levels of activity might serve as positive examples and improve motivation and group cohesiveness. Therefore, considerable intragroup heterogeneity was also accepted.

This study had been approved by the Ethics Committee of the German Society of Physiotherapy (Deutscher Verband für Physiotherapie, 2016-06) and was retrospectively registered in the German Clinical Trials Register (DRKS00010595). It followed the ethical principles of the World Medical Association’s Declaration of Helsinki in its most recent version of Fortaleza. As far as applicable, the reporting adheres to the Consolidated Standards of Reporting Trials (CONSORT) guidelines and to the Template for Intervention Description and Replication (TIDieR) checklist and guide [63]. Funding was provided by the German Federal Ministry for Education and Research (BMBF SILQUA-FH 03FH008SA5).

### Intervention

The intervention occurred in the city of Bochum in the Ruhr metropolitan area (situated in the state of North Rhine-Westphalia in Germany). With its six municipalities, each subdivided into several urban districts, Bochum is particularly affected by population aging [10]. In two initial project meetings, a consortium consisting of the leading project team and representatives from the cooperating organisations – the University of Applied Sciences Ruhr West, the Municipality of Bochum (Social and Health Departments), the Diakonie Bochum (regional welfare association), the Municipality of Bottrop (urban development) and the Fraunhofer Institute for Software and Systems Engineering – selected an eligible municipality. Based on its geographic characteristics, sociodemographic structure, options for and access to activities, the urban district of Bochum-Altenbochum (4.3 km^2^, 12,100 inhabitants) was chosen to host the *Quartier Agil* intervention.

Potential participants were informed about the project’s aim, the time scope, as well as the six components and principles of the intervention. The research team announced that attending the weekly group meeting was mandatory, but the degree to which they would attend further individual and group activities and exercises was completely up to them.

After providing their informed consent, the participants received a Google Nexus 5X Android smartphone, running with a prepaid SIM card and an app specifically designed for *Quartier Agil,* which they were free to use until the end of the intervention cycle. In the first few weeks, the participants received detailed instructions on how to use the smartphone and the *Quartier Agil* app. To be able to align cognitive and physical group activities with the participants’ preferences and habits, relevant and highly frequented sites in the neighbourhood (hot spots) were planned to be identified within the first month based on a group discussion with the participants during the weekly multimodal group training described further below, backed up by tracking their smartphone GPS signals.

The programme was envisaged to exploit the complex and bidirectional relations between social participation, physical and cognitive functions and health through multiple pathways [5, 6] by the implementation of physical, cognitive and social activities, guided by participation tutors (hereafter, tutors). These tutors were charged with instructing and guiding the participants towards the goal of maintaining and enhancing their social participation. The tutors were intended to be healthcare professionals capable of coaching older adults. In this trial, the two tutors comprised a physiotherapist/sport scientist (Master of Arts in Sports Gerontology) and a clinical linguist (Master of Arts).

The tutors should be able to implement and deliver the programme, as well as guide and support the participants through the range of activities. Beyond their professional expertise, they were required to have didactic skills, know effective networking strategies and be experienced with older adults. Their role and their relationship with the participants were planned to vary, depending on the programme component. For complex and challenging physical exercise and cognitive training sessions or for the individual assessment of cognition or physical function, they should provide clear professional guidance and expert advice. For sessions that particularly focused on social interaction or on the participatory advancement of the *Quartier Agil* programme, they should hold back and take a moderating role.

*Quartier Agil* offered six components, from which each participant could choose his/her individually preferred combination (Table 1). The combination of physical and cognitive contents, social activities and smartphone support was supposed to arouse the participants’ interest and ensure a variety of options for them.

**Table 1:** Components of the *Quartier Agil* intervention

	Key elements	Objective	Setting	Tutors’ role	Location	Resources
Multimodal group training	Combined physical and cognitive (dual task) training, education	Improve cognitive and physical resources for relevant everyday functions; promote an (inter-)active lifestyle	Group	Instructing	Gym of the local sports club	Smartphones, training equipment
“Hot spot” activities	Activities in exploring the local environment	Be active; meet people in the neighbourhood; (re-)discover one’s habitat, strengthen link to (social) neighbourhood	Group	Accompanying	“Hot spots” in the neighbourhood	Smartphones, local environment and facilities
Group challenge tasks	Tasks created by group members, posed to (sub-)groups	Be active; create curiosity; improve self-efficacy	Group	Stimulating	Neighbourhood	Dependent on task; mostly smartphones, local environment and facilities
Solitary physical training	Individually tailored physical exercises	Improve physical function	Individual	Supporting	Home	Smartphones, app
Solitary cognitive training	Individually tailored cognitive exercises	Improve cognitive function	Individual	Supporting	Home	Smartphones, app
Personal challenge tasks	Individual cognitive or physical tasks embedded in everyday activities	Be active; create curiosity; improve self-efficacy	Individual	Stimulating	Neighbourhood, home	Dependent on task; mostly smartphones, local environment and facilities

The multimodal group training was scheduled to be held for 1.5 hours once a week on the same day and place. It served to organise the group, introduce the members to the smartphone and the app, gather feedback, perform (dual-task) exercises, and to provide context as to how physical and cognitive abilities change with age and which strategies might be applied to slow down physical and cognitive decline. On the hot spots identified earlier in the process, group activities (e.g., a visit to the weekly market) were scheduled every second week, ideally at the suggestion of the participants as the experts in their own neighbourhood. Additional social and sociocultural group activities (e.g., preparing a meal together, playing bocce in the park) were arranged by the tutors early in each intervention cycle. The group members were then encouraged to take over and self-organise their activities or challenge one another to join public activities, freely choosing the frequency and duration, content and place.

For an additional option, some participants were offered to hold smartphone-supported home-based physical or cognitive training sessions using the respective features of the specifically designed and programmed *Quartier Agil* smartphone app, involving exercise training instructions (videos with strength and balance exercises) and cognitive games (e.g., memory exercises, anagram, Stroop tasks, and word search puzzle). These physical or cognitive tasks were linked to the calendar feature of the *Quartier Agil* app, and the participants were reminded via text messages. Other features of the *Quartier Agil* app included communication (chat feature), and the option to locate other participants in the quarter (which could be turned off by the participants). The individual content and progression of the physical and cognitive training features were managed by the tutors using a web interface. This interface also allowed the tutors to set up quizzes based on QR codes. Lastly, the participants were encouraged to set themselves personal challenge tasks relating to (everyday) physically or cognitively challenging activities, considering their individual needs and aims (e.g., walking instead of driving to the baker’s shop, keeping the shopping list in mind instead of writing it down).

A particular focus of the intervention was the promotion of social participation through the maintenance and/or improvement of (instrumental) activities of daily living, physical mobility and autonomy. If requested, the tutors gave limited advice on possible primary or secondary care based on their therapeutic background and experience, but they did not provide any curative or therapeutic care.

Based on the recommendations by the American College of Sports Medicine [64], the following weekly physical activities and exercises and progression were recommended to most of the participants:

- Aerobic activities: 1 × 20 minutes (first month) ➔ 2–3 × 30–60 minutes (sixth month)
- Resistance training: 1 × 4–6 exercises, 1 × 10–15 repetitions, each with low to moderate resistance (first month) ➔ 1–2 × 4–6 exercises, 3 × 8–12 repetitions with high resistance (sixth month)
- Balance training: 1 × 4 static exercises, 2 × 15–30 seconds each (first month) ➔ 2 × 6 static and dynamic exercises, 4 × 30–60 seconds each or 10–15 repetitions, respectively (sixth month)
- Stretching according to individual needs

During the multimodal group training, for each of these domains, exemplary activities and exercises were performed together, and individual feedback was provided to the participants. Participants were instructed to try out various ways to accumulate the recommended dose of physical activity in each domain throughout every week. In doing so, they were free to choose “hot spot” activities, group challenge tasks, solitary physical training (smartphone-supported), or personal challenge tasks or any preferred combination of these (see Table 1). If participants already met or surpassed the recommended dose of physical activity, they were suggested to slowly increase the intensity or difficulty level, or the total amount of activities.

Cognitive-linguistic training was partially based on existing training programmes (SimA, NeuroVitalis) [37, 38] and progressed according to the following scheme:

- First–sixth month: at least 10 minutes/day, self-paced cognitive training (attention, working memory, executive control, verbal ability), according to individual needs
- First–second month: 30 minutes/week, attention and focus (psychoeducation, group activity)
- Third–fourth month: 30 minutes/week, executive control (psychoeducation, group activity)
- Fifth–sixth month: 30 minutes/week, sensory systems and visual perception (psychoeducation, group activity)

To introduce the cognitive-linguistic exercises, these were first performed in the multimodal group training. Similar to the physical activities, participants could then choose their preferred way to achieve the recommended cognitive-linguistic training dose, e.g. real-life group activities or group challenges; individual, smartphone-supported cognitive training; and/or setting up personal challenge tasks.

During the 6 months of implementation, the content of the training sessions became more complex, and physical and cognitive-linguistic elements were linked. For example, initially, during a stepping task, the participants were asked to adjust their stepping pace and direction according to the appearance of cue words in a story read to them. At the end, the participants practised a rather complex line dance.

### Individual tailoring

During the group training sessions, common criteria were applied to individually tailor the intervention, such as rating of perceived exertion, lack of concentration, as well as signs of exhaustion, pain and/or dizziness. The participants were instructed to reduce the intensity or take a break accordingly. Variations of exercises were offered (e.g., varying the initial body position, number of repetitions) to prompt individual adjustments. If the nature of the task permitted it, the participants were encouraged to help their peers.

The level of difficulty of the app-based cognitive tasks was automatically regulated by individual performance.

### Data collection/assessments

The programme’s feasibility was evaluated at the project level (process, resources and lessons learned) and the participant level (clinical data: physical and cognitive fitness, activity/mobility, participation). Defined sets of quantitatively measured outcome parameters for physical and cognitive fitness, as well as qualitative and self-estimated measures for social participation were used to estimate the magnitude of the effects. Because of the exploratory nature of the study and because of the expectation of a relatively high initial level of physical, cognitive and social activities of the participants, outcomes were not pre-defined as primary, secondary, or tertiary (deviating from the CONSORT statement).

The data on physical functionality and physical capacity, cognitive functionality and cognitive capacity, physical activity, quality of life and social participation were collected before (baseline) and after each of the two six-month cycles of intervention by the tutors (follow-up). The feedback of the participants and the tutors was gathered every fourth week and towards the end of each intervention cycle. The latter included requests and suggestions for upcoming sessions. During the programme, the attendance rate for the various components was recorded by the tutors.

It was planned that the *Quartier Agil* smartphone app also included a documentation feature, capturing login, sensor and self-report data*.* It should allow the participants (and by extension in an aggregated form, the tutors) to log the frequency and duration of app usage, as well as the frequency, amount, type and intensity of the solitary physical training and physical leisure activities, while safeguarding individual participant privacy and fully adhering to the data security requirements in Germany.

#### Physical function and capacity

The six-minute walk test (6MWT) was used to estimate aerobic (endurance) capacity. The 6MWT measures the maximum distance that the participants are able to walk on a flat surface within 6 minutes. While it has been described as sufficiently reliable, valid and responsive (minimum clinically important change: 20 m), ceiling effects do occur in persons with a high aerobic capacity [65].

The Berg Balance Scale (BBS) was used to assess balance and risk of falling. The BBS involves a standardised rating of the duration and quality of the performance in 14 balance-related tasks [66].

Leg strength was assessed by measuring the maximum isometric force of the knee extensors of the dominant leg, according to the Physiological Profile Assessment (PPA), using a digital force gauge attached to the subject’s leg in a standardised seating position (90° knee and hip flexion). The greatest force out of three trials was recorded [67].

#### Cognitive function and capacity

The Montreal Cognitive Assessment (MoCA) was used to screen memory recall abilities, visuospatial abilities, multiple aspects of executive function, attention, concentration, working memory, language and orientation to time and place [62]. It also served to assess exclusion criteria.

The Nürnberger-Alters-Inventar (NAI; Nuremberg Gerontopsychological Inventory) served to evaluate cognitive functions, such as attention, memory and inhibition [68].

The Regensburg Word Fluency Test (RWT) was used to assess cognitive-linguistic performance through word-generation tasks for the evaluation of executive functioning and word fluency [69].

#### Physical activity

For the assessment of habitual physical activity, the participants wore an accelerometer (Actigraph wGT3X) during their waking hours for 1 week. The device was positioned on the hip to register triaxial acceleration at 100 Hz. The data collection and evaluation followed published recommendations [70], using cut-off points for moderate to vigorous physical activity (MVPA), according to Freedson et al. [71]. During that week, the participants were additionally asked to complete a handwritten activity diary and to subjectively estimate their average daily physical activity.

#### Quality of life

The participants rated their health-related quality of life (HRQOL) using the well-established Short Form 12 (SF-12) questionnaire. Based on the 12 questions on the SF-12, physical and mental component summary scores were calculated, each between 0 and 100 (respectively for the lowest and the highest levels of HRQOL) [72].

Additionally, the participants were asked to answer questions relating to their autonomy (AUT); past, present and future activities (PFF); and social participation (SOP) using the respective facets of the World Health Organization Quality of Life–OLD (WHOQOL-OLD) questionnaire [73]. For each facet, the transformed scale score, ranging from 0 to 100, was calculated.

#### Adverse events

Throughout the programme, adverse events, such as falls, cardio-respiratory complications, dizziness/vertigo and injuries of the musculoskeletal system, were documented and classified according to the degree of severity (minor, moderate, serious). Potential causality between the intervention or the assessments and moderate or serious events was analysed post hoc by examining the course of events and circumstances and looking for a plausible link.

#### Social participation

After the six-month intervention, the participants were asked to rate the change of their social participation using self-developed questions. Before and after the six-month intervention, for one week each, the participants were requested to record the number of persons with whom they had contact, the frequency of going outside their homes, the number of social contacts meaningful to them and the number of social contacts with whom they were satisfied.

#### Feedback of participants and tutors

At the end of each cycle, the participants provided structured feedback on the organisation of the programme and their overall satisfaction with it. Feedback was obtained by a questionnaire containing open-ended and closed-ended questions at the follow-up assessments, and a group discussion in one of the last multimodal group training sessions led by the tutors during which the key statements were summed up and noted. The tutors recorded their own impressions regarding the programme’s feasibility and social effects, as well as the participants’ active involvement and behaviour throughout the programme. They were also asked to reflect on the group size and constellation, resources for and barriers to the implementation of the various components, usability and maintenance of the smartphone and apps, as well as the intervention content and scheduling.

### Statistical analysis

For the analysis of the clinical data, an intention-to-treat (ITT) approach was followed, with the last value carried forward in case a participant missed the follow-up test (assuming that the data were missing at random). To perform intragroup comparisons of clinical outcome measures before and after the intervention, the Wilcoxon-Wilcox test was used (IBM SPSS 22). No adjustments were made for covariates. The effect size (ES) *r* was calculated as $r=\frac{\mathrm{Z}}{\sqrt{\mathrm{N}}}$, where Z is derived from the Wilcoxon-Wilcox W test statistic, and N denotes the number of included observations [74].

## Results

### Participants

**Fig. 1:**
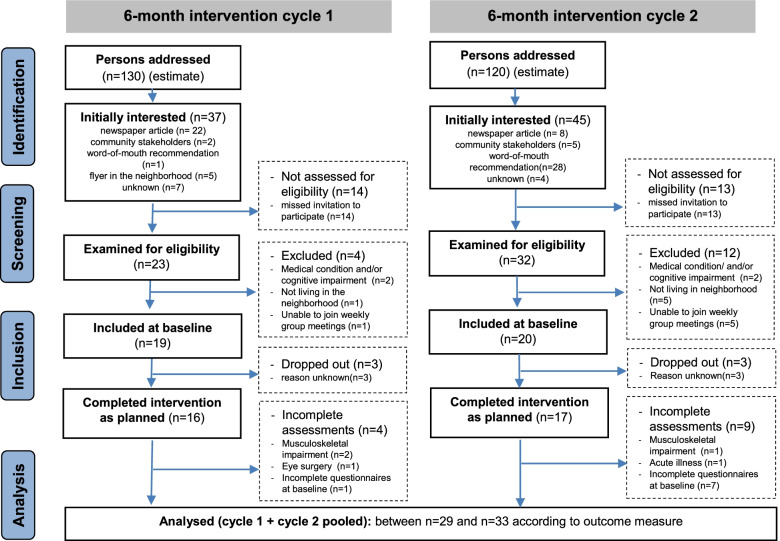
STROBE flow of participants in the subsequent Cycles 1 and 2

Of the potential participants recruited through newspaper advertisements, flyers on relevant sites in the neighbourhood (e.g., cafés or shops), community stakeholders and word-of-mouth recommendations, 19 and 20 persons met the inclusion criteria and were respectively included in Cycles 1 and 2 of the study (Fig. 1).

Descriptive sociodemographic data are presented in Table 2.

**Table 2:** Characteristics of the study sample at baseline (that is, at the start of each 6-month intervention cycle)

	Total (*n* = 39)	Cycle 1 (*n* = 19)	Cycle 2 (*n* = 20)
	Mean (Standard Deviation) or absolute number	Mean (Standard Deviation) or absolute number	Mean (Standard Deviation) or absolute number
Age, years	73.1 (6.8)	71.9 (7.1)	73.6 (6.3)
Body mass index, kg/m^2^	24.7 (4.4)	25.4 (3.2)	24.4 (3.7)
Gender (female)	33	16	17
Fall episode within last 1.5 years	24	17	7
Medical conditions present			
- neoplasms	3	2	1
- endocrine, nutritional and metabolic	11	3	8
- nervous system	3	2	1
- eye	3	0	3
- blood and circulatory system	15	9	6
- musculo-skeletal system	24	11	13

Data are presented as mean ± standard deviation or in absolute numbers

At baseline, the participants accumulated 158 ± 43 minutes of light activity and 30 ± 25 minutes of MVPA per day, with 13 of 32 participants following physical activity recommendations (> 30 minutes of MVPA/day) [75]. With a mean of 493 ± 74 meters, 25 of 33 participants performed better in the 6MWT than the age- and gender-specific norm at baseline [76]. Seventeen of 33 participants scored higher in the BBS than their age- and gender-specific norm value at the baseline [77]. MoCA scores indicated no clinically relevant case of cognitive impairment [61] in the sample (Table 4).

### Recruitment

The two tutors spent a total of 20 work hours on making preparations and recruiting participants (31 minutes per successfully recruited participant). The eligibility criteria were highly applicable, and there were no unclear cases. The eligibility criteria did not seem too restrictive, with an estimated recruitment rate (proportion of persons included at baseline from the persons initially addressed – see Fig. 1) above 10%.

### Assessments

The two tutors spent a total of 26 hours on making preparations and organising the baseline and follow-up assessments (20 minutes per participant). Actually, conducting the assessments took 164 hours in total (approximately 2 hours per participant). For various reasons, the assessments were partly incomplete for 13 of 33 participants (Fig. 1).

### Attendance during intervention

In total, six participants dropped out during the intervention period (Fig. 1). The average attendance rate in the weekly group training (offered 24 times per cycle) was 76 ± 15%. In each cycle, almost half of the participants performed 10 additional activities at hot spots (e.g., a photo safari, during which individually meaningful landmarks were to be photographed and then shared with the group; a bike ride along the “Springorum” bicycle path on a former mine railway track; a teatime quiz at the well-known traditional “Wickenburg” bakery) and 3 group challenge tasks (e.g., playing bocce or Kubb; “mental walks” where routes were first to be imagined, and then actually walked).

### Intervention: resources and implementation

The two tutors were both present at all the group sessions and in most additional activities that they had organised, except for holidays and solitary individual training. They each spent approximately 6 hours per week during Cycle 1 and 3 hours per week during Cycle 2 on the conception and implementation of all activities (excluding smartphone support).

For the weekly multimodal group training serving as an anchor, the gym of a local sports club was rented. This facility provided a broad range of sports equipment and training materials (Swiss ball, stepper, small sandbags, mats, etc.), which were complemented by weight bands and board and throwing games.

The amount of time that was initially planned for smartphone training in the weekly group sessions turned out to be too short and had to be extended. Furthermore, it had to be supplemented by individual support. Introducing the smartphones to the participants and supporting them, adjusting settings, assisting in solving technical problems and coordinating further problem solving with the technical partners required an extra 3 hours in the initial weeks of each cycle and approximately 1 hour towards the end of each cycle for each tutor.

The hot spot activities and the group challenge tasks were offered in the neighbourhood as planned. The smartphone was employed as an essential element of many of these activities. The exercise training and QR code scanner features of the Android app specifically designed for *Quartier Agil* were deemed useful, whereas its communication feature turned out to be of little use. Therefore, the latter feature was soon replaced with a standard app (WhatsApp), which was widely used for intragroup communication during and after each intervention cycle.

### Intervention: example

An example of the actual programme offered in a typical week of *Quartier Agil* (week 13 of 25) is shown in Table 3. More detailed information will be made available in German on https://www.hs-gesundheit.de/forschung/abgeschlossene-projekte/quartier-agil.

**Table 3:** Sample week of the *Quartier Agil* programme

Day	Monday	Tuesday	Wednesday	Thursday
**Component**	**Solitary cognitive training**	**Solitary physical training**	**Multimodal group training**	**Hot spot activities**
Content	Cognitive game Anagram via app	Six strength exercises via app	Greeting administration Cognitive game Homonym Obstacle course + cognitive task outlook	Paper chase: “On the trail of …” hike through botanical garden, combined with a smartphone-based quiz
Duration	5 minutes	30 minutes	90 minutes	60 minutes
Time	At the discretion of each participant	At the discretion of each participant	10:30–12:00	Self-organised by a number of participants
Location	Home	Home	Local sports club	Botanical garden
Mentoring	–	–	2 tutors	–
Resources	Smartphone, app	Smartphone, app	Word list, cones, hoop, balance pads	Smartphone, quiz
Organised by	1 tutor	1 tutor	2 tutors	2 tutors

In this example, no activity was scheduled from Friday to Sunday. The content of the multimodal group training and the actual hot spot activities usually changed from week to week

### Intervention: modifications

Based on the participants’ feedback and on the tutors’ experiences, as planned, the intervention was modified after completing Cycle 1.

Because of the challenges and the time expenditure related to the smartphone training and support, this was trimmed down to cover only the most relevant functions. Basic features, such as the messaging app or the contact information of other group members, were pre-installed. Furthermore, the tutors offered a fixed, clearly limited time slot prior to each group session for the introduction to specific functions, individual consultation and troubleshooting. The tutors also paired advanced smartphone users with less experienced ones and asked the participants to support one another. The time gained was utilised for physical and cognitive exercises, as well as for facilitating group activities and getting to know one another.

For a further change in Cycle 2, the tutors included more didactic elements to promote group processes and social networking. They applied more games for familiarisation, promoted practising in small groups or in pairs and frequently changed seating arrangements. They also repeatedly let the group reflect on and discuss the project aims. Exercises, activities and app features that were rated ‘poor’ after Cycle 1 were replaced by other items. For example, instead of a modified ball game, a net-stepping task was provided to the participants of Cycle 2.

Most of the recurring technical issues with the smartphones were resolved by either the technical partners or the tutors using non-digital workarounds. This can be illustrated by the smartphone tracking module that was intended to identify highly frequented sites in the neighbourhood (hot spots) and to interconnect participants in real time (i.e., while they were moving around in the neighbourhood). Due to problems with the GPS signal processing and filtering, this tracking and interconnection module did not work as intended. As a workaround, hot spots were then identified based on a simple paper-and-pencil alternative and on a discussion with all participants to align group activities with their preferences and habits.

### Intervention: participants’ feedback

The participants’ feedback indicated good overall satisfaction with the programme, and most participants asked for an extension of the intervention period. Of 29 participants, 27 stated that their expectations had been fulfilled. Twenty-two of 29 participants clearly felt that the programme had indeed helped them make new contacts and expand their social network, as well as actively participate in the social life of their neighbourhood. While this was not formally evaluated, the participants’ smartphone competency seemed considerably improved.

Of the group activities, the QR code-based quiz in the nearby botanical garden, a relay of 100 questions and the throwing game Kubb were rated most positively. Modified ball games and other sports games were less popular.

The smartphone videos to guide individual strength and balance training were initially regarded as helpful but were actually used to varying degrees. Cognitive challenges offered via the *Quartier Agil* app were appreciated and used after some time. The overall satisfaction with the specifically designed app varied between participants because of both dispensable and missing features. However, after the initial barriers had been overcome, the smartphone itself and the conventional messaging app were lauded and used quite frequently.

### Intervention: tutors’ feedback

Generally, the tutors rated *Quartier Agil* as a feasible programme. However, they addressed some limiting factors. Extensive differences in cognitive and physical capabilities among the participants, as well as varying extrinsic motivators and expectations, made it difficult to design and deliver an intervention that was suitable and interesting for everyone. Due to this heterogeneity, the group size (20 persons) was regarded as the maximum, which still enabled safe and tailored supervision by the two tutors. The smartphone introduction and support were perceived as the greatest challenge to this project. Abundant minor and major technical problems and delays consumed the time spent with the group and required intensive cooperation with the technical partners, relating to the front-end, back-end and data processing components. Finally, albeit to a lesser degree in Cycle 2, the tutors thought that the participants were hesitant to show initiative in co-producing their activities. While they became familiar with each other and got involved over the course of each cycle, they were still slow to get active themselves. So, even towards the end, they relied on the participation tutors to bring in ideas instead of designing the individual and the group challenges on their own.

### Adverse events

There were neither moderate nor serious adverse events during the investigation period that could be traced back to the intervention or assessments of the *Quartier Agil* project.

### Preliminary estimate of effects

Most outcome measures (except improved balance) related to physical function and physical activity did not significantly differ between baseline and follow-up (Table 4). Because of problems with the data collection using the *Quartier Agil* app and because of a large number of inconsistent entries in the activity diary, the actual types of activities could not be analysed.

**Table 4:** *Quartier Agil* intervention effects

		Baseline		Follow-up		Wilcoxon-Wilcox test (W)		Effect size
	*n*	*Median (Range)*		*Median (Range)*		*z*	*p*	*r*
Berg Balance Scale (score)	33	54 (41–56)		55 (44–56)		−2.33	.020	.41
	*n*	*Mean (Standard Deviation)*	*95% CI*	*Mean (Standard Deviation)*	*95% CI*	*z*	*p*	*r*
Six-minute walk test (m)	33	493 (74.3)	467–519	500 (82.9)	470–529	−1.05	.295	.18
Isometric strength: knee extension (kg)	33	21.6 (7.32)	19.0–24.2	22.2 (9.00)	19.0–25.4	- 0.38	.705	.07
Light physical activity (% of wear time)	32	18.8 (5.44)	16.8–20.8	18.7 (5.16)	16.9–20.6	−0.11	.909	.02
Moderate to vigorous physical activity (% of wear time)	32	3.94 (3.34)	2.74–5.14	3.80 (2.95)	2.74–4.87	−0.07	.943	.01
MoCA (score)	33	25.7 (2.20)	24.9–26.5	25.7 (2.47)	24.8–26.6	−0.14	.890	.02
NAI subtest: figure test (*n* remembered items)	33	5.27 (1.01)	4.92–5.63	5.64 (0.78)	5.36–5.91	−2.03	.042	.35
NAI subtest: wordlist (sum *n* copied and recognised items)	33	5.73 (2.11)	4.98–6.48	6.12 (1.73)	5.51–6.73	−1.09	.274	.19
NAI subtest: numbers connection test (RT in s)	33	25.3 (12.1)	21.1–29.7	22.5 (5.94)	20.4–24.6	−0.47	.638	.08
NAI subtest: numbers symbol test (score)	33	47.9 (9.66)	44.4–51.3	48.42 (9.47)	45.1–51.8	−0.52	.603	.09
NAI subtest: Stroop (interference score, RT in s)	33	23.5 (11.9)	19.3–27.7	20.2 (9.17)	17.0–23.5	−2.18	.030	.38
NAI-Subtest: latent learning (*n* remembered items)	33	6.09(1.40)	5.59–6.59	6.24 (1.09)	5.86–6.63	−0.47	.641	.08
RWT subtest: formal lexical verbal fluency	33	62.4 (21.4)	54.8–70.0	70.4 (23.5)	62.0–78.7	−1.64	.100	.29
RWT subtest: formal lexical verbal fluency change	33	63.1 (24.6)	54.4–71.8	75.9 (21.0)	68.4–83.3	−3.25	.001	.57
RWT subtest: semantic verbal fluency	33	75.8 (24.1)	67.3–84.3	83.5 (20.0)	76.4–90.6	−2.75	.006	.48
RWT subtest: semantic verbal fluency change	33	72.2 (24.0)	63.8–80.7	74.0 (24.0)	65.0–82.0	−0.74	.462	.13
SF-12	31							
PCS		48.1 (7.63)	45.3–50.9	46.3 (10.8)	42.4–50.3	−1.05	.296	.19
MCS		24.3 (9.24)	20.9–27.6	24.9 (7.83)	22.0–27.8	−0.04	.970	.01
WHOQOL-OLD	*32*							
AUT		*78.9 (13.7)*	74.0–83.9	*78.9 (13.3)*	74.1–83.7	−0.09	.929	.02
PPF		*74.4 (16.8)*	68.4–80.5	*74.1 (17.0)*	67.8–80.2	−0.22	.830	.04
SOP		*76.2 (16.0)*	70.4–81.9	*77.3 (19.4)*	70.3–84.4	−0.75	.456	.13
	*n*	*Absolute number*		*Absolute number*		*z*	*p*	*r*
Activity diary	29							
Contact persons/week						*0*	1.0	*0*
0 persons		0		0				
1–3 persons		6		4				
4–7 persons		16		20				
8+ persons		7		5				
Left home/week						*−1.41*	.157	*.26*
0 times		1		0				
1–3 times		25		25				
4–7 times		3		4				
8+ times		0		0				
Meaningful contacts/ week						*−0.71*	.48	*.13*
0 contacts		0		0				
1–3 contacts		17		17				
4–7 contacts		12		10				
8+ contacts		0		2				
Satisfaction with contacts						*0*	1.0	*0*
very high		9		9				
high		15		14				
moderate		4		6				
low		1		0				
not satisfied		0		0				

*Abbreviations*: *MoCA* Montreal Cognitive Assessment, *NAI* Nuremberg Gerontopsychological Inventory, *RT* reaction time, *n* quantity, *RWT* Regensburg Word Fluency Test, *PCS* physical component summary score, *MCS* mental component summary score, *AUT* Autonomy, *PPF* past, present and future activities, *SOP* social participation

Cognitive testing revealed a significantly improved short-term memory from baseline to follow-up and an increased inhibition, as well as a significant improvement of executive functions and word fluency, displayed by two of the word generation tasks of the RWT (Table 4). All other cognitive parameters showed a trend towards consistent or more efficient cognitive functioning but did not reach significance.

Quality of life and indicators of social participation did not significantly differ between baseline and follow-up (Table 4).

## Discussion

According to this exploratory proof-of-concept study, the *Quartier Agil* approach to promote older people’s social participation in their neighbourhood seems feasible and safe. Its core components and principles (provide and enable social interaction, take place in the neighbourhood, involve participants and use digital resources) can be included and followed, albeit to varying degrees and at different costs. Adaptations in the intervention and in the assessments may contribute to better assessment and exploitation of the potential of the *Quartier Agil* approach.

### Resources needed

More resources than anticipated were necessary for the recruitment and assessments, as well as for the implementation of some components of the intervention.

The high number of programme components and the consideration of perspectives from multiple disciplines, such as sociogerontology and psychogerontology, prevention sciences, and sports and therapy science, strengthen the programme and contribute to a truly bio-psycho-social approach. However, in practice, this increases the complexity and the resources needed, which becomes particularly apparent in developing and organising the smartphone support.

#### Recruitment

The recruitment was sufficiently feasible. For future studies, it is interesting to note that in the first cycle, at a time when we did not have any personal connections to stakeholders or potential participants in the neighbourhood, most participants were recruited via newspaper advertisement. Later, as soon as some personal relationships were established (Cycle 2), most participants were recruited by word-of-mouth recommendations. The smartphone support was an important attractor for programme participation. This also had an impact on how the group was composed and what the preferences of the participants were later in the programme. It is unclear if less cognitively, physically and/or socially active persons (which would be the actual target group in a future larger study) could be recruited with similar ease. This may require different recruitment strategies.

#### Intervention

Overall, with the changes introduced for Cycle 2 (as listed under the sub-heading Intervention: Modifications), the intervention was feasible. Nonetheless, several adaptations could be considered to strengthen it.

Introducing the smartphone and apps to the participants turned out to be very time-consuming for the tutors. In this situation, more and better organised interactions among group members may improve less experienced users’ adoption of the new technology. Taking advantage of the heterogeneous group constellation by pairing experienced and inexperienced users in Cycle 2 turned out to be useful, together with restricting time for smartphone lessons. For the experienced participants, helping and instructing less skilled group members seemed to solidify their own knowledge, while the less experienced participants indeed seemed to increase their digital competency because of this help [78, 79]. For the future, another option might be to involve digital natives (e.g., younger relatives, such as grandchildren).

#### Assessments

Baseline and follow-up assessments could be performed safely and were generally feasible, but some participants had incomplete data. The total number of assessments may have overwhelmed these participants, as further discussed in the subsection “Adequacy of outcome parameters”. In the future, the relevance of these assessments (and of the completeness of the data) could be explained more carefully to the participants (e.g., by additionally providing them with a video), and all data should be immediately checked for completeness and plausibility (enabling further enquiry if needed).

### Participant satisfaction and lessons learned

Overall, *Quartier Agil* was well received, with few dropouts. Specific adjustments may be useful based on the feedback of the participants and the tutors, as listed in the Results section. The most important adjustments likely concern the smartphone app and the participants’ views on their own role in the project.

The app was specifically designed and programmed for *Quartier Agil*, intended to ease or enable goal and incentive setting, and offering home-based physical or cognitive training sessions. Another planned feature was the documentation of physical training, activities and visited locations, and most importantly, older people’s social interaction itself. However, some of the features of the app were not well received by the participants. Further, some of the documentation features were not (completely) programmed because of the limited resources available, while other features did not function properly, or were unreliable. We had underestimated the iterative process of app development and the recurring bugs that could not to be resolved quickly. Underlying problems included the front-end, back-end and data processing components of the sensing system.

For future interventions, the research team would aim to either select established and user-tested apps well before the start of the project (commercial, open-source, prototype) or plan to use paper-and-pencil alternatives. As an example, WhatsApp successfully initiated brisk communication among the participants far beyond the *Quartier Agil* intervention. Studies on instant messaging (e.g., WhatsApp) provide some evidence of a positive relation between computer-mediated communication and perceived social support and connectedness among community-dwelling older adults [80]. With ongoing technological development, smartphone apps will offer more options and may better adhere to standards for mobile health-related apps. However, it will still be challenging to identify suitable, affordable and easy-to-use applications for specific means, such as goal setting, describing behavioural patterns over time (documentation of activities/ visited locations) and examining social network systems.

Researchers could attempt to better explain the nature of social interaction to the participants and to further gather and discuss their individual perceptions of social participation. This would consume more time early on and might require better use of didactic strategies, but it could result in a greater or a more specific commitment of the participants and allow them to co-produce the intervention instead of just perceiving themselves as recipients. Encouraging participants’ exchange on the specific objectives of the project could be more explicitly extended to the underlying concept of enhancing activity and cognitive function as a means to enable participation, while stressing the relation between physical-cognitive functions and (perceived) social participation as individual and varying among persons. Researchers could also better illustrate to participants that (training) activities are not only means of maintaining health but equally represent a form of autonomy, participation and social interaction themselves.

### Adequacy of outcome parameters

The data collection and testing provided valuable information on several parameters relevant for social participation. The participants perceived it as quite extensive, so the researchers did not add any assessments in Cycle 2. However, the outcomes may not have fully covered the scope of the interventions (e.g., concurrent motor and cognitive task training, and smartphone education and training) and the aims of *Quartier Agil* (e.g., to prevent both physical decline and social isolation in the neighbourhood, and to increase health behaviour competence). Outcomes such as dual-tasking abilities [81], perceived competence and confidence to handle and use a smartphone [82], the size of the personal life space [83] and physical activity-related health competence [84] might offer advantages over some of the rather clinical and functional outcomes chosen for this feasibility study. Dual-tasking abilities, smartphone competence, life space and health behaviour competence might better reflect the multidimensionality of the researchers’ approach, have higher relevance for or be more closely linked to participation and be more sensitive to the actual intervention.

Such outcomes should not simply be added to the current set but would need to replace existing functional outcomes. The choice of parameters to assess both the process and the outcomes should ultimately be guided by a refined theory of change (logic model) of the *Quartier Agil* intervention, while strengthening the focus on social participation. The social relationships of the present study’s participants should be characterised in more detail to be able to reflect on and understand the implications of their different nature and type. For example, in previous studies, friendship ties were found to be more closely related to late-life physical activity than family ties [85].

In this context, the utility of a detailed quantitative collection of social interactions, daily activities and mobility patterns – with the goal of actually guiding the intervention – needs to be discussed critically. While using (big data) analytical techniques might help expose relevant behaviour patterns and (inter-)relations with social participation during the intervention, the sole reliance on objective data might detract participants from finding and communicating their own perspectives and solutions. As a technological solution, sensor data might be used to trigger context-contingent, ecological momentary assessments [86], which could later be used for scientific analysis, as well as assist in individual and group reflections. For example, when a participant changes his/her location in the neighbourhood (obtained via GPS data), an app might immediately deliver related questions (e.g., “Why are you here?” “Whom are you with?” “Do you feel that you are socially participating?”). Participant incentives might be necessary to encourage the use of the sensing app for long periods of time.

Focus group discussions, semi-structured interviews and photovoice should complement the quantitative assessments. Apart from producing scientific findings, these qualitative assessments could also be viewed as a participatory method and part of the intervention, since these might contribute to triggering more thorough reflection and communication processes regarding social participation, the participants’ role in the project and their neighbourhood, among others.

### Magnitude of effects

According to this study’s results, *Quartier Agil* may safely contribute to the preservation of physical functioning and the improvement of cognitive functioning that are relevant for participation and health in an already physically and cognitively active population. While some of these effects’ relevance for older persons can hardly be underestimated, and some individuals informally reported considerable real-life impacts, these functional effects had a limited magnitude on a group level. Due to the broad inclusion criteria resulting in a relatively fit yet heterogeneous group, and because of the accentuated multimodal nature of the programme, this finding was somewhat expected.

Unfortunately, the actual amount of individual training and activity throughout the six-month intervention remains unclear, since the self-reported extent and intensity of the solitary physical training, as well as the usage/login data for the *Quartier Agil* app features including the cognitive training were either not available in time, or were apparently not logged correctly. Using more reliable and user-friendly logging technology may allow researchers to analyse possible dose-response relations in future studies.

### Future study

A larger controlled study to more comprehensively investigate the *Quartier Agil* approach seems warranted. However, in order to be able to actually detect effects, this future study should focus on older adults who are considerably less active than the participants in the current trial. Further, the intervention and the outcome assessments should be revised according to the suggestions offered in the preceding sections.

### Limitations

This study was deliberately not set up to have high internal and external validity. Its purpose was to systematically gather information on the feasibility of both the *Quartier Agil* intervention approach and a study to evaluate this approach. This study’s limitations include its non-controlled design; the small, selective convenience sample, which is not representative of the actual target group; and the relatively high number of incomplete assessments. The lack of exclusion criteria regarding current physical and cognitive activities should be considered as a particularly significant limitation.

## Conclusions

Overall, the *Quartier Agil* approach to promote autonomy and social participation of older people living in the same neighbourhood through combined physical and cognitive training, supported by technical devices, such as smartphones, appears feasible. Even for the physically and cognitively active and capable population, modest improvement or at least the preservation of physical and cognitive functions relevant for participation could be observed. Likewise, the approach offers older people, who have different interests and motives, the opportunity to have a sense of achievement and to gain experience on a variety of levels (social participation, digital participation, technical competence, physical function, cognitive capacity and connectedness to the neighbourhood). Increasing solidarity and exchange among neighbourhood residents may contribute to community-building and enhanced neighbourhood cohesion.

The variety of overlapping social, digital, cognitive and physical activities may contribute to making this unique programme interesting and attractive to diverse participants. However, such a broad range of intervention components necessarily comes at a price, that is, the portion of each component and its effect on the corresponding domain may be limited. Furthermore, the multimodal nature of the project and the use of smartphones (including the app) require a significant organisational effort and tie up considerable resources for its supervision.

Based on the results presented in this paper, a future larger study to assess the effects of a refined *Quartier Agil* intervention on social participation as a primary outcome, and revised secondary outcomes among socially, physically and cognitively less active elderly persons seems worthwhile.

Meantime, this study’s approach and the lessons learned have generated substantial interest among the people involved in the project and other municipalities and neighbourhoods. Even in its current (preliminary) conceptualisation, the project may provide interesting options for sports clubs, municipalities, local charities, churches, welfare organisations and other social institutions to extend the existing engagement and their presence in the neighbourhood. The recent concept of the BMBF-funded project *Quartier Agil* is described in more detail in a German manual, which will be made available (for application in other neighbourhoods) at https://www.hs-gesundheit.de/forschung/abgeschlossene-projekte/quartier-agil.

## Data Availability

The datasets used and analysed in this study are available from the corresponding author on reasonable request.

## Abbreviations

6MWT: six-minute walk test
AUT: autonomy facet of the WHOQOL-OLD questionnaire
BBS: Berg Balance Scale
BMBF: Federal Ministry of Education and Research
FUNTEX: Functional Task Exercise
HIFE: High Intensity Functional Exercise
HRQOL: health-related quality of life
ITT: intention-to-treat
LIFE: Lifestyle Functional Exercise Program
MoCA: Montreal Cognitive Assessment
MVPA: moderate to vigorous physical activity
NAI: Nuremberg Gerontopsychological Inventory
PFF: past, present and future activities facet of the WHOQOL-OLD questionnaire
PPA: Physiological Profile Assessment
RWT: Regensburg Word Fluency Test
SF-12: Short Form 12
SIMA: Maintaining and supporting Independent Living in Old Age
SOP: social participation facet of the WHOQOL-OLD questionnaire
TIDieR: Template for Intervention Description and Replication
WEBB: Weight-Bearing Exercise for Better Balance
WHOQOL-OLD: World Health Organization Quality of Life–OLD
